# The *what* and *why* of perceptual asymmetries in the visual domain

**DOI:** 10.2478/v10053-008-0080-6

**Published:** 2010-12-15

**Authors:** A. K. M. Rezaul Karim, Haruyuki Kojima

**Affiliations:** 1 Department of Psychology, University of Dhaka, Bangladesh; 2 Graduate School of Human and Socio-environment Studies, Kanazawa University, Japan

**Keywords:** visual perception, asymmetry, within-visual field, between-visual field, primary why, critical why, neural mechanisms, hemispheric specialization, visual experience

## Abstract

Perceptual asymmetry is one of the most important characteristics of our visual functioning. We carefully reviewed the scientific literature in order to examine such asymmetries, separating them into two major categories: within-visual field asymmetries and between-visual field asymmetries. We explain these asymmetries in terms of perceptual aspects or tasks, the *what* of the asymmetries; and in terms of underlying mechanisms, the *why* of the asymmetries. Tthe within-visual field asymmetries are fundamental to orientation, motion direction, and spatial frequency processing. between-visual field asymmetries have been reported for a wide range of perceptual phenomena. foveal dominance over the periphery, in particular, has been prominent for visual acuity, contrast sensitivity, and colour discrimination. Tthis also holds true for object or face recognition and reading performance. upper-lower visual field asymmetries in favour of the lower have been demonstrated for temporal and contrast sensitivities, visual acuity, spatial resolution, orientation, hue and motion processing. Iin contrast, the upper field advantages have been seen in visual search, apparent size, and object recognition tasks. left-right visual field asymmetries include the left field dominance in spatial (e.g., orientation) processing and the right field dominance in non-spatial (e.g., temporal) processing. left field is also better at low spatial frequency or global and coordinate spatial processing, whereas the right field is better at high spatial frequency or local and categorical spatial processing. All these asymmetries have inborn neural/physiological origins, the *primary why*, but can be also susceptible to visual experience, the *critical why* (promotes or blocks the asymmetries by altering neural functions).

## INTRODUCTION

Visual perception is the process of interpreting and organizing visual information. It involves our ability to recognize and identify the distinguishing features of visual images such as shape, size, orientation, position, colour, etc. We have very powerful vision and visual perception, with many surprising properties. One of the most prominent properties is the perceptual variability or asymmetry resulting from the brain’s preferential responses to some visual stimuli and/or to stimuli at some specific retinal location. For example, vertical stimuli are perceived and represented better than oblique stimuli (e.g., Campbell, Kulikowski, & Levinson, 1966; Furmanski & Engel, 2000; Mitchell, Freeman, & Westheimer, 1967), sensitivity to foveal stimuli is stronger than to peripheral stimuli (e.g., Duncan & Boynton, 2003; Hansen, Pracejus, & Gegenfurtner, 2009; Virsu & Rovamo, 1979), etc. Over the last few decades, researchers have identified dozens of phenomena exhibiting perceptual asymmetries that may not be apparent in our conscious awareness while we are accomplishing everyday tasks. Yet we still do not have an account that gives a comprehensive global picture of perceptual asymmetries and their emergence. This review is, therefore, an in-depth analysis of the perceptual asymmetries in visual psychophysics and visual neurology that have been documented thus far.

A careful look at previous research in these two areas indicates that, while some information is processed quickly or efficiently, some information may be delayed or processed less efficiently within the same location of the visual field. On the contrary, when information in a particular visual field location, say, central, is efficiently processed it may be poorly processed in the opposite location (here, peripheral). Thus, perceptual asymmetries in the visual domain can be separated into two major categories: within-visual field (WVF) asymmetries and between-visual field (BVF) asymmetries. In this review, we term the perceptual aspects or tasks in which asymmetries appear the *what* of the asymmetries and the underlying mechanisms the *why* of the asymmetries. The *why* of perceptual asymmetries can be further divided into the *primary why* and the *critical why*. The *primary why* refers to the physiological mechanisms or neural organizations we are born with. The *critical why*, on the other hand, refers to the experiential or learning factor that interacts with the *primary why* and thereby changes the organizational and functional features of cortical neurons. However, for ease of comprehension the what and the *primary why* of the asymmetries are explained together, and followed by an explanation of the *critical why*.

## THE *WHAT* AND *PRIMARY WHY* OF THE WITHIN-VISUAL FIELD ASYMMETRIES

### Asymmetry in orientation processing

Visual perception exhibits many examples of anisotropic behaviour, where the percept’s relationship to the stimulus changes with the orientation of the stimulus. Specifically, psychophysical studies have shown that performance is better at the cardinal than the oblique orientation in contrast sensitivity (Campbell & Kulikowski, 1966; Mitchell et al., 1967), stereoacuity (Mustillo, Francis, Oross, Fox, & Orban, 1988), grating acuity (Berkley, Kitterle, & Watkins, 1975; Campbell et al., 1966), and vernier acuity (Corwin, Moskowitz-Cook, & Green, 1977; Saarinen & Levi, 1995; Westheimer & Beard, 1998). This fact, often referred to as the oblique effect (Appelle, 1972), is most prominent in central vision (e.g., Berkley et al., 1975; Mansfield, 1974).

The oblique effect is functionally important as V1 (primary visual cortex/striate cortex) neurons are organized into orientation columns (Hubel & Wiesel, 1968, 1974a). This has also been confirmed in later single-neuron electrophysiological (DeValois, Yund, & Hepler, 1982; Li, Peterson, & Freeman, 2003), optical imaging (Coppola, White, Fitzpatrick, & Purves, 1998), and fMRI (Furmanski & Engel, 2000) studies. For example, an fMRI study has demonstrated that grating acuity is finer for cardinal (horizontal and vertical) than for oblique stimuli (Furmanski & Engel). This study also reveals a corresponding asymmetry in neural populations in V1, that is neural responses in V1 are greater for cardinal than oblique stimuli.

In addition to the oblique effect, scientists have demonstrated “horizontal-vertical” asymmetry in a variety of visual tasks. For example, our contrast sensitivity and spatial resolution are better along the horizontal mid-line of the visual field than along the vertical mid-line (Carrasco, Talgar, & Cameron, 2001; Rijsdijk, Kroon, & van der Wildt, 1980). This is consistent with the fact that within the cardinal more cells are devoted to horizontal than vertical orientation (Li et al., 2003). Thus, the orientation asymmetries have a primarily physiological or neural basis.

Very recently Karim and Kojima (2010, in press) have demonstrated that, within a specific orientation performance in vernier offset detection may vary as a function of vernier configuration (spatial arrangement of light bars). In one study, they have claimed that vernier offset detection at the cardinal orientation depends on the relative position of the vernier features (Karim & Kojima, 2010). Specifically, for a pair of horizontal light bars (vernier features) arranged side-by-side with a large gap between them observers were, on average, better at discriminating a vertical offset if the right-hand bar was below the left-hand bar than vice versa. Similarly, for a pair of vertically oriented light bars, one above the other, the horizontal offset detection was better if the lower bar was on the left of the upper bar rather than on its right. In another study, they have shown that this effect can be generalized to the oblique orientation (Karim & Kojima, in press). They concluded that these asymmetries might be due to neural preference or selectivity for one particular vernier configuration rather than another and that such preference possibly developed through early experience or through evolution (Karim & Kojima, in press).

### Asymmetry in motion processing

Meridian-dependent effects (oblique effects) have also been found in our perception of moving objects (e.g., Coletta, Segu, & Tiana, 1993; Loffler & Orbach, 2001; Matthews & Qian, 1999). In general, psychophysical studies have concentrated on the anisotropy of the precision in motion direction discrimination. In contrast to motion detection thresholds, which have been found to be isotropic (e.g., Raymond, 1994; Van de Grind, Koenderink, Van Doorn, Milders, & Voerman, 1993), motion discrimination thresholds depend on the absolute direction of motion. This meridian-dependent anisotropy for direction of motion discrimination has been reported for random dots (e.g., Ball & Sekuler, 1982; Gros, Blake, & Hiris, 1998) as well as for translating plaids composed of a couple of gratings (Heeley & Buchanan-Smith, 1992). These anisotropies have been observed either in foveal vision or in a visual space at specific eccentricity (i.e., within a specific location in the visual field).

Electrophysiological studies show that within an orientation column of the V1, cells share a similar preferred orientation but they have diverse physiological properties, one of the most dramatic being direction selectivity (cf. Gur, Kagan, & Snodderly, 2005; Hubel & Wiesel, 1968). That is, at any preferred orientation neurons that are direction selective in V1 respond more strongly to one direction of motion than to the opposite direction (Pasternak, Schumer, Gizzi, & Movshon, 1985; Reid, Soodak, & Shapley, 1991). The signal is then dispatched via the near extrastriate V2 and V3 to the far extratriate V5/MT (middle temporal) for a further analysis (e.g., Albright, 1984; Britten, Shadlen, Newsome, & Movshon, 1992). Numerous studies have shown that neurons of similar orientation or direction-of-motion selectivity are clustered into functional columns in the MT (Albright, Desimone, & Gross, 1984; Diogo, Soares, Koulakov, Albright, & Gattass, 2003; Malonek, Tootell, & Grinvald, 1994). In addition, a very large proportion of MT neurons are selective for the direction of motion and the orientation of moving gratings (Born & Bradley, 2005). Using the stimuli of moving gratings, a recent optical imaging study of owl monkeys has demonstrated that more of the MT cortical space is devoted to representing cardinal than oblique orientation (Xu, Collins, Khaytin, Kaas, & Casagrande, 2006), the anisotropy being more prominent in central vision (≤ 10°). Furthermore, neural responses to cardinal orientation were greater than neural responses to oblique orientation. It has been claimed that this data explains why there is greater sensitivity to motion discrimination when stimuli are moved along the cardinal meridians (polar axes), suggesting that the motion oblique effect either originates in the MT or is enhanced at this level (Xu et al.).

### Asymmetry in spatial frequency processing

The primate V1 is dominated by complex cells that respond preferentially not only to orientation and motion direction but also to the spatial frequency (SF) of the stimuli (DeValois & DeValois, 1988). Human psychophysical studies suggest that there is a continuous distribution of the SF preference in the visual cortex. For example, observations in SF-specific adaptation (Blakemore & Campbell, 1969) and SF discrimination (Watson & Robson, 1981) tasks provide compelling evidence that the visual cortex has multiple processing channels, each tuned into one of many different SF ranges (Sachs, Nachmias, & Robson, 1971; Watson, 1982). In accord with this, electrophysiological studies have shown that V1 neurons have a wide range of SF preferences (DeValois, Albrecht, & Thorell, 1982; Tolhurst & Thompson, 1981) and neighbouring neurons are more likely to prefer similar SFs (DeAngelis, Ghose, Ohzawa, & Freeman, 1999; Maffei & Fiorentini, 1977; Tolhurst & Thompson, 1982).

SF preference also appears in later stages of visual processing, the degree of preference being varied across the visual areas. For example, animal studies have shown that SF preference is higher in V1 than in extratriate V2 (Foster, Gaska, Nagler, & Pollen, 1985; Issa, Trepel, & Stryker, 2000) and V3 (Gegenfurtner, Kiper, & Levitt, 1997). This fact has also been confirmed in a recent fMRI study of humans (Henriksson, Nurminen, Hyvärinen, & Vanni, 2008).

These studies have demonstrated the SF preference either in central vision or in a visual space at specific eccentricity (i.e., within a specific location of the visual field). However, such preferences are more pronounced in the central vision and decrease with eccentricity.

## THE *WHAT* AND *PRIMARY WHY* OF THE BETWEEN-VISUAL FIELD ASYMMETRIES

### Foveal versus peripheral asymmetries

Perceptual capacity depends on where stimuli are located in the visual field. Something we see out of the corner of our eye is blurred until we turn our eyes to look directly at it. This is partly due to the sparse distribution of cones in the periphery and partly due to the neural structures of the visual cortices. That is, the density of the receptors in our visual system decreases as distance from the fovea increases (e.g., Curcio, Sloan, Kalina, & Hendrickson, 1990; Curcio, Sloan, Packer, Hendrickson, & Kalina, 1987). This lack of uniformity carries through to lateral geniculate nucleus (Connolly & Van Essen, 1984) and into visual cortices in both human (e.g., Anstis, 1998; Qiu et al., 2006; Sjöstrand, Olsson, Popovic, & Conradi, 1999) and non-human primates (e.g., Hubel & Wiesel, 1974b; Maunsell & Van Essen, 1987; Van Essen, Newsome, & Maunsell, 1984). Consequently, visual acuity (DeValois & DeValois, 1988; Duncan & Boynton, 2003), contrast sensitivity (Virsu & Rovamo, 1979), and colour detection/discrimination (e.g., Hansen et al., 2009; Mullen, 1991; Mullen & Kingdom, 1996; Newton & Eskew, 2003) fall significantly towards the periphery. Many other visual functions such as object and face identification (Rousselet, Thorpe, & Fabre-Thorpe, 2004), stereopsis and reading are also essentially limited to the central visual field (Battista, Kalloniatis, & Metha, 2005; Zegarra-Moran & Geiger 1993). Consequently, the visual cortex’s early selective response towards stimuli such as faces declines dramatically if presented a few degrees away from the fovea or central fixation (Eimer, 2000; Jeffreys, Tukmachi, & Rockley, 1992).

### Upper versus lower visual field asymmetries

Visual performance degrades in the periphery of the visual field, but not proportionately in the lower and upper fields. Typically, the lower visual field supports better performance than the upper visual field, even at the same eccentricity (Danckert & Goodale, 2001; Levine & McAnany, 2005; McAnany & Levine, 2007). Psychophysical studies have demonstrated the dominance of the lower field in temporal and contrast sensitivities (Skrandies, 1987), visual acuity (Skrandies, 1987), spatial resolution (Rezec & Dobkins, 2004), and in hue (Levine & McAnany, 2005) and motion (Edwards & Badcock, 1993; Lakha & Humphreys, 2005; Levine & McAnany, 2005; Raymond, 1994) processing. This phenomenon is known as the vertical meridian asymmetry, which becomes more pronounced with eccentricity (Carrasco et al., 2001) and with increased spatial frequency. It is barely present for low spatial-frequency Gabor stimuli, and gradually becomes more pronounced for intermediate and high spatial frequencies (Carrasco et al., 2001; Liu, Heeger, & Carrasco, 2006; Skrandies, 1987). Many studies have also reported that the lower field advantages may be restricted to the vertical meridian (polar axis that runs from above the observer’s line of sight, through the fixation point, and to below the observer’s line of sight) and may not be observed in non-meridian locations (Carrasco et al., 2001; Liu et al., 2006; Talgar & Carrasco, 2002).

Neurophysiological studies have confirmed the advantages of the lower visual field’s sensitivity to contrast patterns (Portin, Vanni, Virsu, & Hari, 1999), high contrast checkerboards (Fioretto et al., 1995), non-attended colour changes (Czigler, Balazs, & Pato, 2004), and motion (Kremláček, Kuba, Chlubnová, & Kubová, 2004). In addition, this sort of measure has revealed the advantages of the lower hemi-field over the upper hemi-field, indicating that the asymmetry is not specific to the vertical meridian as opposed to the psychophysical reports described above. Specifically, non-human primate studies have shown that the cone and ganglion cell densities in the retina are greater for the lower than for the upper visual field (Perry & Cowey, 1985). Slightly more neural tissue is devoted to the lower than the upper visual field representations in LGN (lateral geniculate nucleus; Connolly & Van Essen, 1984), V1 (Van Essen et al., 1984), and MT (Maunsell & Van Essen, 1987). Human electrophysiological studies have also indicated functional specialization for the lower and upper visual fields. For example, visual 100 ms evoked potential peaks 11 to 12 ms earlier for lower visual field stimuli than for upper visual field stimuli (Lehmann & Skrandies, 1979; Skrandies, 1987). Similarly, MEG response amplitude has been reported to be greater for the lower than the upper visual field in human observers (Portin et al., 1999). All this evidence for processing differences and functional effects concerns eccentricities greater than around 5° (Portin et al.). Thus, it is unclear whether the processing of visual information differs between the lower and upper visual fields near the fovea.

### Left versus right visual field asymmetries

Perceptual processing in the left and right visual fields depends on the spatiality of stimulus. Typically, spatial information is processed more precisely in the left visual field and non-spatial information in the right visual field (Boulinguez, Ferrois, & Graumer, 2003; Corballis, 2003; Corballis, Funnell, & Gazzaniga, 2002; Okubo & Nicholls, 2008). Specifically, the left visual field is better at simple line orientation, vernier offset and size discriminations (Corballis et al., 2002), and the right visual field at temporal (Okubo & Nicholls, 2008), linguistic and cognitive processing (Corballis, 2003). The superiority of the left visual field is explained by the right hemisphere (RH) dominance over the left hemisphere (LH) in spatial attention, as demonstrated in studies with healthy individuals (Heilman & Van Den Abell, 1979; Sturm, Reul, & Willmes, 1989) as well as in unilateral lesion (Mattingley, Bradshaw, Nettleton, & Bradshaw, 1994), and neuroimaging studies (Corbetta, Miezin, Shulman, & Peterson, 1993). On the other hand, presentation of verbal (i.e., non-spatial) stimuli produces left-hemisphere activation, which triggers a rightward attentional bias and results in a right visual field advantage (Cohen, 1982; Kinsbourne, 1970). Furthermore, an in-depth analysis of previous research reveals that spatial processing in the left and right visual fields can be different in many ways, as illustrated below.

#### High versus low spatial frequency processing

Visual analytic skill is critically determined by the stimulus spatial frequency (SF), depending on its location in the visual field. The stimuli with low SF are processed more efficiently in the left visual field and those with high SF are processed more efficiently in the right visual field. This asymmetric processing is directly associated with the functional specialization of the RH and LH, which correspond to the left and right visual fields, respectively. That is, the RH is dominant for low SF processing whereas the LH is dominant for high SF processing. Evidence of this hemispheric specificity has been provided by psychophysical and behavioural studies using gratings of different spatial frequencies (Christman, Kitterle, & Hellige, 1991; Kitterle, Hellige, & Christman, 1992; Kitterle & Selig, 1991) and natural pictures of low and high spatial frequencies (Peyrin, Chauvin, Chokron, & Marendaz, 2003; Peyrin et al., 2006). Recent functional brain imaging studies conducted on healthy participants also support this pattern of functional cerebral organization (Peyrin, Baciu, Segebarth, & Marendaz, 2004; Peyrin et al., 2005).

#### Global versus local processing

As global and local stimuli are typically conveyed by low SF and high SF, respectively, global information is processed more efficiently in the left visual field and local information in the right visual field (cf. Grabowska & Nowicka, 1996; Ivry & Robertson, 1998; Sergent, 1982). For example, reaction time to a global target is faster than to a local target when stimuli are presented in the left visual field, and vice versa when they are presented in the right visual field (Hübner, 1997; Kimchi & Merhav, 1991; Sergent, 1982). This global versus local processing asymmetry has been confirmed in neuropsychological (lesion) studies (e.g., Delis, Robertson, & Efron, 1986; Hickok, Kirk, & Bellugi, 1998; Lamb, Robertson, & Knight, 1989), and in other behavioural studies with healthy humans (e.g., Blanca, Zalabardo, Gari-Criado, & Siles, 1994; Hübner, 1998; Versace & Tiberghien, 1988; Yovel, Yovel, & Levy, 2001).

In line with this, neuroimaging studies have shown that global and local perception is mediated by separate subsystems in the RH and LH, respectively. For example, ERPs (Event-related potentials) to compound stimuli presented at the central fixation induce a larger occipito-temporal negativity (Heinze, Johannes, Münte, & Magun, 1994; Schatz & Erlandson, 2003) or target-specific difference waves (Han, Liu, Yund, & Woods, 2000; Proverbio, Minniti, & Zani, 1998) over the RH in global stimulus condition, but over the LH in local stimulus condition. Similarly, PET and fMRI studies have shown increased regional cerebral blood flow or hemodynamic responses in the right lateral occipital cortex when attending to the global structure of compound stimuli, but in the left occipital cortex when attending to the composing local elements (Fink et al., 1996; Han et al., 2002; Martinez et al., 1997).

#### Coordinate versus categorical processing

 Visual processing depends on how the stimulus elements are spatially related in the visual display. Kosslyn (1987) theorized that the visual system uses two types of spatial relations: coordinate relations and categorical relations. Coordinate relations specify precise spatial locations of objects or object parts in terms of metric units and give exact distances. Categorical relations, in contrast, assign a spatial configuration or a range of positions to an equivalence class (e.g., above/below, left/right, inside of/outside of) without defining the exact metric properties. In the last couple of decades, scientists have reported visual field asymmetry in processing these kinds of dual spatial relations. In particular, categorical spatial processing is better in the right visual field and coordinate spatial processing is better in the left visual field (Hellige & Michimata, 1989; Kosslyn et al., 1989). For example, Hellige and Michimata (1989) had participants judge if a dot was above or below a line (a categorical task) or judge if the dot was within 2 cm of the line (a coordinate task). A right visual field advantage was present for the categorical task and a left visual field advantage was present for the coordinate task. Kosslyn (1987) has attributed this kind of fact directly to the functional specialization of the two hemispheres. He proposed that the LH is preferentially associated with the between-item categorical processing (e.g., one item is “above” or “below” the other, a discrete judgment) and the RH is preferentially associated with the between-item coordinate processing (e.g., one item is “near” to or “far” from the other, an analog judgment). These distinct types of spatial processing may also occur for the relative positions of parts or features of a single stimulus item (Slotnick & Moo, 2006). 

 Kosslyn’s model, that there are two types of spatial representations each with a specific lateralization pattern, has received some support in different lines of behavioural research (Banich & Federmeier, 1999; Laeng & Peters, 1995; Michimata, 1997; Niebauer, 2001; Okubo & Michimata, 2002; Rybash & Hoyer, 1992; Wilkinson & Donnelly, 1999). However, some attempts to replicate the findings have failed (Bruyer, Scailquin, & Coibion, 1997; Sergent, 1991a, 1991b), though no studies have yet found the reverse pattern of hemispheric dissociation. Procedural differences might explain this failure of replication. For example, a study conducted by Bruyer et al. (1997) suggests that the hemispheric dissociation for categorical and coordinate processing is highly unstable and sensitive to subtle methodological factors, which could preclude its general application. In that study, Kosslyn’s hypothesized dissociation was observed in the manual requirement but not in the oral response requirement, in the feedback but not in the no-feedback condition, and in younger but not in elderly observers. Kosslyn’s hypothesis should, therefore, be carefully tested employing similar stimuli and procedures so that a firm conclusion about the existence of hemispheric dissociation for coordinate and categorical processing can be arrived at. 

 Besides the two categories of perceptual asymmetries, there is evidence of top-left lighting prior in 3D shape discrimination task that does not fall in either of the categories. Half a century earlier, Gestalt psychologist Metzger (1936) noticed that left-lit scenes have greater perceptual value than right-lit ones. His observations gave rise to an intriguing possibility: The visual system assumes that light is coming from the left-above rather than straight-above. Sun and Perona (1998) have tested this proposition by asking observers to look for a convex or concave object lit from one direction among similar objects lit from the opposite direction. In this study, the shaded targets are detected more quickly when the illumination position is between 30° and 60° to the left of vertical. Both left- and right-handed participants show this tendency, but it is more pronounced among the right-handed. This preference also occurs in artists, participants across schools and periods of art history, indicating its ecological significance. The top-left lighting preference has been supported in a number of studies (e.g., Gerardin, de Montalembert, & Mamassian, 2007; Mamassian, Jentzsch, Bacon, & Schweinberger, 2003), with the difference that it may not be associated with handedness (Mamassian & Goutcher, 2001). For example, in a recent study of localization of an odd part of the Polo Mint stimulus Gerardin et al. (2007) found better performance for the stimuli lit from the left than from the right. In another study of shape from shading, Mamassian et al. (2003) detected a stronger top-left preference when the stimulus is presented foveally rather than para-foveally. These authors have also claimed that the N2 and P1 components in the visual occipital and temporal areas might be responsible for the preference towards the leftward lighting position, thus indicating a neural basis for the phenomenon. However, there is still no evidence that this preference can be associated with hemispheric specialization. Hence, the phenomenon is unspecified in this review. 

## VISUAL EXPERIENCE: THE CRITICAL WHY OF PERCEPTUAL ASYMMETRIES

As discussed above, the asymmetric processing of visual information has either a physiological or a neural basis. One of the most conspicuous functional properties of neurons in the visual cortex is orientation selectivity, as more cortical circuitry represents cardinal orientations rather than oblique orientations. The development of this feature is primarily under endogenous control (e.g., Chapman, Stryker, & Bonhoeffer, 1996; Coppola & White, 2004; Wiesel & Hubel, 1974), but can also be altered by visual experience, sometimes with dramatic effects (e.g., Blakemore & Van Sluyters, 1975; Crair, Gillespie, & Stryker, 1998; Sengpiel, Stawinski, & Bonhoeffer, 1999). Specifically, an early electrophysiological study of monkeys (Wiesel & Hubel, 1974) and a recent optical imaging study of ferrets (Coppola & White, 2004) have demonstrated that overrepresentation of cardinal orientations in the visual cortex does not require experience of an anisotropic visual environment. Visual experience is not necessary for the initial development of cortical orientation maps. Early maps are seen in ferrets’ visual cortices before natural eye opening (Chapman et al., 1996), and normal orientation maps develop in kittens that have been binocularly deprived for the first three weeks of their lives (Crair et al., 1998). However, longer periods of binocular deprivation cause degradation of orientation preference maps (Crair et al., 1998), indicating that visual experience is necessary for their maintenance. This is consistent with prior electrophysiological (Blakemore & Van Sluyters, 1975) and later optical imaging results (Sengpiel et al., 1999). The development of orientation selectivity does not require visual experience, but is critically dependent on spontaneous neuronal activity (Chapman, Gödecke, & Bonhoeffer, 1999; Coppola & White, 2004). Absence of normal visual experience can block spontaneous neural activity and hence orientation selectivity.

 Unlike orientation selectivity, the development of direction selectivity requires visual experience. Li, Fitzpatrick, and White (2006) investigated the development of direction selectivity in ferrets’ visual cortices using optical imaging and electrophysiological techniques. In their study, direction selectivity was detected several days after eye opening, this strengthened to adult levels over the following 2 weeks. Visual experience was essential for this process, as shown by the absence of direction selectivity in dark-reared ferrets. The impairment persisted in dark-reared ferrets that were given experience of light after this period, despite the recovery of orientation preference. Similarly, a recent study has shown that the visually naïve ferrets’ visual cortices exhibited a well defined system of orientation columns, but lacked the columnar pattern of direction selective responses (Li, Hooser, Mazurek, White, & Fitzpatrick, 2008). These results provide strong evidence that visual experience increases the magnitude of direction selectivity, but there was no change in orientation selectivity after training. The researchers concluded that early experience with moving visual stimuli drives the rapid emergence of direction selective responses in the visual cortex. Thus, visual experience is necessary for the development of direction selectivity, as opposed to the development of orientation selectivity. We suggest that the development of direction selectivity and orientation selectivity are two independent processes of the visual system, the former being experience-bound while the latter is not. In addition, direction selective cortical neurons may be more susceptible or vulnerable to early visual experience than orientation selective neurons. 

Visual experience, or learning, also has a crucial role in modifying the response properties of higher cortical neurons. Numerous neurophysiological studies provide evidence for after-training enhanced stimulus selectivity. In particular, neurons in monkey IT (inferior temporal) cortices show enhanced selectivity after training for novel objects (Kobatake, Wang, & Tanaka, 1998; Rolls, 1995), holistic multiple-part configurations (Baker, Behrmann, & Olson, 2002), and even physically unrelated pairs of shapes (Messinger, Squire, Zola, & Albright, 2005). The time required for response changes in some of these neurons parallel the time required for learning (Messinger, Squire, Zola, & Albright, 2001), suggesting a strong link between underlying neuronal plasticity and behavioural improvement. Furthermore, learning can shape the assignment of novel objects into classes (Rosenthal, Fusi, & Hochstein, 2001). This shaping is done by modulating the selectivity of neurons in the inferior temporal and frontal cortex for features crucial for the categorization process (Freedman, Riesenhuber, Poggio, & Miller, 2006). A couple of studies have, however, reported that more PF (prefrontal) neurons (Rainer & Miller, 2000) or more V4 neurons (Rainer, Lee, & Logothetis, 2004) are selective towards repeated rather than novel stimuli at a moderate level of image degradation, and at undegradation the effect was reverse or equivalent. This indicates that stimulus selectivity is not a stable property of cortical neurons; rather it is sensitive to the context of stimulation. We suggest that response preference varies not only across the stimulated areas of the visual cortex, but also within a particular area depending on the context of the stimulation. However, in order to reach a firm conclusion regarding this trend the hypothesis should be experimentally addressed in all other visual areas.

Psychophysical studies have also shown that visual experience modifies visual response. For example, a 3D shape discrimination study has demonstrated that the visual system’s prior knowledge or assumption about the direction of lighting (i.e., “light-from-above” prior) can be modified by visual-haptic training in humans (Adams, Graf, & Ernst, 2004). This study has shown that training affects not only subsequent shape perception of trained stimuli but also generalizes to affect the perceived reflectance of novel stimuli. The effect has been successfully replicated in a recent study on shape discrimination (Champion & Adams, 2007). In addition, Champion and Adams have shown that convexity prior in visual search tasks (Langer & Bülthoff, 2001) can be reduced by training. These findings suggest that cortical neurons learn where light-sources are usually located, as well as the actual shape of the object, from interactions with the environment, and use this information to interpret subsequent visual stimuli.

In their recent studies Karim and Kojima (2010, in press) have shown that vernier acuity improves as a function of training in both the cardinal and oblique orientations. In addition, configurational asymmetry in vernier acuity reduces more or less with training in the cardinal (Karim & Kojima, 2010) but not in the oblique orientation (Karim & Kojima, in press). This indicates a cardinal versus oblique orientation difference in sensitivity to training. They interpreted this fact by the same mechanism of the oblique effect. That is, a much lower percentage of V1 neurons are tuned to the oblique than to the cardinal orientation (Coppola et al., 1998; DeValois et al., 1982; Furmanski & Engel, 2000; Li et al., 2003). In addition, neurons with the oblique preferences exhibit wider orientation tuning widths than neurons with the cardinal preferences (Kennedy & Orban, 1979; Nelson, Kato, & Bishop, 1977; Orban & Kennedy, 1981; Rose & Blakemore, 1974). Thus, the asymmetry might reduce with training at a slower rate in the oblique than in the cardinal orientation.

Taking all these results together, visual experience can modify or shape the response properties of cortical neurons that contribute to perceptual asymmetries. Thus, visual experience (or learning) can play a critical role (promoting or hindering) in perceptual asymmetry.

## SOME UNRESOLVED AND CONTRADICTORY ISSUES

In spite of the successful demonstration of visual perceptual asymmetries in various dimensions, there remain a number of contradictory and unresolved issues in the scientific literature. As part of the above review, the cardinal superiority of visual performance over the oblique orientation directly corresponds to the asymmetry of neural organization in the primary visual cortex. But, it is still unclear why visual acuity is finer for vertical stimuli than it is for horizontal stimuli (Saarinen & Levi, 1995), a demonstration opposed to the fact that more cells are devoted to horizontal than to vertical orientation (Li et al., 2003). Some scientists have tried to associate this demonstration with the everyday fact that we experience more vertical than horizontal stimuli (Gregory, 1997). However, we wonder to what extent such normal visual experience can alter the physiological preponderance and why such inconsistency does not occur for some other visual tasks such as contrast sensitivity and spatial resolution (Carrasco et al., 2001; Rijsdijk et al., 1980). That is, our daily experience may not be responsible for altering typical orientation asymmetry. In fact, the orientation detectors and many other feature detectors in the two hemispheres are neither functionally equivalent nor are they absolutely exclusive or independent. So, we propose that any consistency/inconsistency between our orientation perception and cell representation can be determined by the balanced/imbalanced functional interaction of the two hemispheres rather than by normal visual experience.

 A more important topic that requires scientific attention is that all the previous studies of visual experience (see above) are concerned with how experience or learning shapes the WVF asymmetries. No attempt has been made to explore whether this factor can also accelerate or hinder the BVF asymmetries. For example, the left-right visual field asymmetries have been associated with the functional specializations of the corresponding hemispheres, but no attention has been paid to whether experience can alter their specialized functions. In the case of the upper-lower visual field asymmetries the lower field advantages have been interpreted by finer attentional resolution in the lower than in the upper visual field (He, Cavanagh, & Intriligator, 1996, 1997; Intriligator & Cavanagh, 2001), but we still do not know whether this increased attentional resolution is learned or innate. We suggest that it might be learned, at least partly, because we usually look downward rather than upward in our daily activities. However, past scientific studies cannot give a satisfactory explanation of the superiority of the upper field over the lower field in visual search (Previc & Blume, 1993), apparent size (Ross, 1997), and object recognition tasks (Chambers, McBeath, Schiano, & Metz, 1999) tasks. In fact, these observations are contradictory to the demonstration that attentional resolution is finer (He et al., 1996, 1997; Intriligator & Cavanagh, 2001) and neural representation is larger in the lower than in the upper visual field (Connolly & Van Essen, 1984; Lehmann & Skrandies, 1979; Maunsell & Van Essen, 1987; Portin et al., 1999; Skrandies, 1987; Van Essen et al., 1984). However, a theoretical account of the disparity between upper and lower field dominance was proposed by Previc (1990), referring to the differences between the two major streams of primate’s visual processing: the subcortical (magnocellular/parvocellular) level (Breitmeyer, 1992) and cortical (dorsal/ventral organization) level (Ettlinger, 1990; Goodale & Milner, 1992; Ungerleider & Mishkin, 1982). Previc (1998) posited that the processing of stimuli from lower and upper visual fields are promoted by these two neural systems, and that they are related to the near (peripersonal) and far (extrapersonal) spaces, respectively. According to this perspective, the specialization of the lower and upper visual fields and their neural systems depends on the segregation of the near and far spaces, which occurred during primate evolution. The lower visual field was mainly involved in the perceptual processes required for visuomotor coordination in the peripersonal space, largely performed by the dorsal pathways of the primate’s visual system. And the upper visual field was linked to the visual search and recognition mechanisms directed towards the extrapersonal space, primarily controlled by the ventral system. However, Previc’s ideas are based on his assumption about primate evolution and lacks empirical support. 

## CONCLUDING REMARKS

This review demonstrates the wide range of perceptual variability or asymmetry both within- and between-visual fields. The within-visual field asymmetries are typically caused by neural preferences or asymmetric neural distribution in visual cortices. However, silencing cortical activity or preventing visual experience may block the typical development of the asymmetries. The foveal-peripheral asymmetries have been attributed to the biased distribution of retinal cones and cortical neurons. The upper-lower visual field asymmetries have been explained by asymmetric neural distribution and attentional resolution, whereas the left-right visual field asymmetries have been explained by stimulus driven attentional bias or by the functional specialization of the two hemispheres. However, it remains unknown whether visual experience can change the BVF asymmetries. Also, it has yet to be investigated whether either hemisphere can independently determine the WVF asymmetries. This would be very challenging because such an attempt would require physiological isolation of the cerebral hemispheres. The two hemispheres of the brain are actually designed to constantly communicate with one another and their separation for experimental purpose may lead to functional abnormality or at least some discrepancies between pre- and post-separation. However, we suggest that as human brains are plastic, like the WVF asymmetries the BVF asymmetries can be modified by visual experience. Empirical confirmation of this hypothesis would enable scientists to alter the functional properties of the hemispheres in the expected direction. This knowledge could be used for human welfare, particularly for the brain-damaged patient population.
